# Is chest X-ray necessary after central venous catheter insertion?

**DOI:** 10.1186/cc13319

**Published:** 2014-03-17

**Authors:** MS Vallecoccia, F Cavallaro, M Biancone, D Settanni, C Marano, MG Annetta, M Pittiruti, M Antonelli

**Affiliations:** 1 Catholic University, Rome, Italy

## Introduction

According to the European Society of Parenteral and Enteral Nutrition guidelines [1], post-insertion chest X-ray (CXR) is not necessary if the location of the tip has been verified during the procedure and if pleura-pulmonary damage has been ruled out by other methods. The aim of this study is to assess feasibility and safety of an echo-ECG-guided method of central venous catheter (CVC) insertion and to evaluate whether post-insertion CXR can be avoided.

## Methods

We enrolled only patients admitted to our ICU and candidate to elective CVC insertion, who had a detectable P wave on surface ECG. Our insertion protocol included: preliminary ultrasound (US) scan of central veins and pleural space; US-guided puncture and US control of the correct direction of the guidewire; intracavitary ECG method for tip location (cavo-atrial junction (CAJ) = maximal P wave); and US scan of pleural space to rule out pneumothorax (PNX). Post-insertion CXR was performed in all patients to rule out PNX and verify tip location close to the CAJ (CAJ = 3 cm below the carina [2]). Tip location between 1 and 5 cm below the carina - in the lower 1/3 of the superior vena cava (SVC) or in the higher 1/3 of the right atrium (RA) - was considered acceptable [1].

## Results

Eighty CVCs were placed in 78 patients, either by residents in training or by attending doctors. The vein was selected by US scan: right internal jugular (IJ) 64%, left IJ 17%, right axillary 11%, left axillary 4%, right innominate 4%. One pre-existing PNX was identified by US before the procedure. Accidental arterial puncture occurred in three cases (two by residents). In five cases, the wrong direction of the wire was detected by US and corrected during the procedure. The mean number of attempts was 1.65 (1.35 for attending vs. 1.95 for residents, *P *< 0.02) and mean insertion time was 10.4 minutes (8.72 for attending vs. 12.11 for residents, *P *< 0.005). Pleural US scan ruled out puncture-related PNX in all patients. At post-procedural CXR: no PNX was detected, 90% of tips were in the target zone, 5% were in the middle 1/3 of SVC and 5% were in the middle 1/3 of RA (all CVC with tips out of the target zone were inserted by residents).

## Conclusion

Our insertion protocol was safe and effective, with minor differences between experienced versus nonexperienced operators. Our data suggest that immediate CXR is not necessary soon after CVC insertion, since PNX can be ruled out by US and since the intracavitary ECG method allows tip location in the SVC and RA in all cases.
